# Serum Vanin-1: a potential diagnostic biomarker linked to oxidative stress imbalance in asthma

**DOI:** 10.1186/s12890-026-04319-7

**Published:** 2026-05-04

**Authors:** Wei Li, Yun Liu, Xiangli Feng, Xiaoli Feng, Xiuyuan Wei, Yuanyuan Wu

**Affiliations:** Department of Respiratory and Critical Care Medicine, The Second Affiliated Hospital of Xi’an Jiaotong University, No.157, West 5th Road, Xi’an, Shaanxi 710004 P.R. China

**Keywords:** Asthma, Vanin-1, Biomarker, Oxidative stress, Inflammation

## Abstract

**Background:**

Asthma is a heterogeneous disease, underscoring the urgent need for reliable diagnostic biomarkers. Vanin-1, a well-recognized sensor of oxidative stress, has been implicated in various inflammatory disorders; however, its diagnostic value in asthma remains to be fully elucidated. This study aimed to evaluate serum Vanin-1 levels in asthmatic patients and explore their correlations with systemic oxidative stress biomarkers—malondialdehyde (MDA) and glutathione (GSH)—as well as inflammatory cytokines, pulmonary function parameters, and to assess its diagnostic potential.

**Methods:**

A case–control study was conducted, enrolling 169 participants: 129 asthmatic patients and 40 age- and sex-matched healthy controls. Serum concentrations of Vanin-1, MDA, GSH, cytokines (IL-4, IL-13, IL-17, IFN-γ), and total IgE were measured. Pulmonary function tests were also performed. Statistical correlations were analyzed using Spearman’s rank test, and diagnostic efficacy was evaluated using receiver operating characteristic (ROC) curves.

**Results:**

Compared with healthy controls, asthmatic patients exhibited significantly elevated serum levels of Vanin-1 (7.54 ± 1.62 ng/mL vs. 4.59 ± 1.30 ng/mL, *P* < 0.001) and MDA [0.11 (0.09, 0.12) nmol/mg protein vs. 0.08 (0.06, 0.10) nmol/mg protein, *P* < 0.001], but markedly reduced GSH [1.44 (1.16, 1.57) nmol/mg protein vs. 2.06 (1.65, 2.37) nmol/mg protein, *P* < 0.001]. Vanin-1 level was positively correlated with MDA (*ρ* = 0.342, *P* < 0.001) and negatively correlated with GSH (*ρ* = − 0.329, *P* < 0.001). No significant correlations were observed between Vanin-1 and IL-4, IL-13, IL-17, IFN-γ, eosinophil counts, or pulmonary function indices. ROC analysis demonstrated that Vanin-1 had robust diagnostic utility, with an area under the curve (AUC) of 0.884 (95% CI: 0.832–0.936, *P* < 0.001). At an optimal cutoff of 6.12 ng/mL, the sensitivity and specificity were 69.0% and 92.5%, respectively.

**Conclusion:**

Serum Vanin-1 is significantly elevated in asthmatic patients and is closely associated with an oxidative stress imbalance. It may serve as a potential biomarker for distinguishing asthma patients from healthy individuals.

**Supplementary Information:**

The online version contains supplementary material available at 10.1186/s12890-026-04319-7.

## Introduction

Asthma is a prevalent chronic inflammatory airway disease characterized by variable airflow obstruction, bronchial hyperresponsiveness, and underlying immune dysregulation [1]. Despite advances in asthma management, its marked heterogeneity continues to pose substantial challenges to accurate diagnosis and personalized therapy, underscoring the need for reliable biomarkers [2, 3]. Oxidative stress is increasingly recognized as a key contributor to asthma pathophysiology, driving airway inflammation and remodeling through an imbalance between reactive oxygen species production and antioxidant defenses [4, 5].

Vanin-1, a glycosylphosphatidylinositol-anchored pantetheinase, has emerged as a critical regulator of oxidative stress and inflammation in various diseases [6]. It catalyzes the hydrolysis of pantetheine to produce cysteamine and pantothenic acid (vitamin B5), thereby directly influencing the biosynthesis of GSH—the primary intracellular antioxidant [7]. Previous studies have established Vanin-1’s role in inflammatory conditions; for instance, its expression and promoter methylation status can discriminate corticosteroid treatment responses in pediatric asthma [8]. Furthermore, Vanin-1 pathway activation correlates with disease severity in systemic sclerosis, where it influences fibrosis, vasculopathy, and oxidative stress [9]. In inflammatory bowel disease, polymorphisms in regulatory regions of the Vanin-1 gene are associated with disease susceptibility, highlighting its importance in mucosal inflammation [10].

As an epithelial pantetheinase, Vanin-1 overexpression is increasingly regarded as a predictor of inflammation-related pathologies [11]. Vanin-1-deficient mouse models exacerbate acetaminophen-induced hepatotoxicity, linked to impaired GSH metabolism and defective immune responses [10]. Accumulating evidence indicates dysregulated Vanin-1 expression across a spectrum of pathological conditions, including diabetes [11], septic shock, kidney injury [12], periodontitis [12], primary nephrotic syndrome [13], cancer [14], and chronic obstructive pulmonary disease (COPD) [15]. Notably, fluctuations in Vanin-1 expression may serve as a marker for tracking disease progression [6]. Vanin-1 contributes to the maintenance of redox homeostasis, as demonstrated by the enhanced oxidative stress tolerance and attenuated inflammatory responses observed in Vanin-1^-^/^-^ mice [16].

However, the clinical value of circulating Vanin-1 as a biomarker in adult asthma remains largely unexplored. Therefore, we hypothesized that circulating Vanin-1 levels are elevated in asthmatic patients. To test this hypothesis, we aimed to: 1) investigate serum Vanin-1 levels in asthmatic patients compared to healthy controls; 2) analyze the relationships between Vanin-1 and systemic oxidative stress markers (MDA, GSH) as well as inflammatory cytokines; 3) evaluate the diagnostic performance of Vanin-1 for asthma.

## Materials and methods

### Study population and recruitment

A total of 129 patients with asthma and 40 age- and sex-matched healthy control subjects were enrolled in this study between January 2024 and January 2025 at the Second Affiliated Hospital of Xi’an Jiaotong University. The study protocol was approved by the Ethics Committee of the Second Affiliated Hospital of Xi’an Jiaotong University (Approval No. 2025 Lun Shen 232). All participants provided written informed consent before enrollment. The detailed inclusion and exclusion criteria for each group are summarized in Table 1.

**Table 1:** Inclusion and exclusion criteria for the asthmatic patient and healthy control groups

	Healthy control group	Asthma group
Inclusion	(1) No obvious abnormality was found in chest X-ray or CT, the results of pulmonary function test were normal (FEV_1_/FVC > 0.70, FEV_1_ > 80% predicted); (2) No history of respiratory diseases such as asthma, chronic obstructive pulmonary disease, pulmonary infection, lung cancer, allergic rhinitis, eczema, GERD and other allergic diseases, and no history of autoimmune diseases. (3) Age 14–75 years old.	(1) Age 14–75 years old; (2) First diagnosed with asthma and strictly conforming to the diagnostic criteria of the Global Initiative for Asthma (GINA) 2023 guidelines (with variable asthma symptoms and positive results of variable airflow limitation tests; for atypical asthma without obvious wheezing, at least one variable airflow limitation test is positive); (3) patients were required to be in a stable phase at the time of enrollment (no asthma exacerbations, emergency room visits, hospitalizations, or systemic corticosteroid use in the 4 weeks preceding enrollment.).
Exclusion	(1) Smoking history: Current smokers or ex-smokers with a smoking history of ≥ 10 pack-years (to rule out smoking-related lung disease) (2) BMI ≥ 28 kg/m^2^	(1) Respiratory comorbidities: Diagnosis of other chronic respiratory diseases (e.g., COPD, bronchiectasis, interstitial lung disease, obstructive sleep apnea, GERD or active tuberculosis); (2) Inflammatory conditions: Active autoimmune diseases, malignancies, or acute infections (viral or bacterial) within the 4 weeks prior to enrollment; (3) Smoking history: Current smokers or ex-smokers with a smoking history of ≥ 10 pack-years (to rule out smoking-related lung disease); (4) Other significant comorbidities: Severe cardiovascular, hepatic, or renal dysfunction; uncontrolled diabetes; or pregnancy/lactation; (5) Recent corticosteroid use: Use of systemic corticosteroids within the past 4 weeks (for asthmatics, this was an exclusion criterion; for controls, any history of corticosteroid use was exclusionary). (6) BMI ≥ 28 kg/m^2^

### Serum biomarker analysis

Peripheral venous blood samples (approximately 5 mL) were collected from all participants after an overnight fast. The samples were allowed to clot at room temperature and then centrifuged at 3000 rpm for 10 min to obtain serum. Aliquots of serum were stored at − 80 °C and analyzed within 1 month.

Serum levels of Vanin-1 and T-helper (Th) cell-related cytokines (IL-4, IL-13, IL-17, and IFN-γ) were quantified using commercially available ELISA kits (Shanghai Sinobestbio Technology Co., Ltd., China; Vanin-1: Cat. No. YX-E23022; IL-4: Cat. No. YX-E10142; IL-13: Cat. No. YX-E10152; IL-17: Cat. No. YX-E10082; IFN-γ: Cat. No. YX-E10162), in strict accordance with the manufacturer’s protocols. Total IgE (tIgE) levels and peripheral blood eosinophil counts were assessed by the clinical laboratory department of the Second Affiliated Hospital of Xi’an Jiaotong University. Specifically, tIgE was measured using the ImmunoCAP Total IgE Anti-IgE reagent (Phadia AB, Thermo Fisher Scientific, Uppsala, Sweden; Cat. No. 52-5461-CN/04) per the manufacturer’s protocol.

MDA levels were quantified using a Thiobarbituric Acid Reactants (TBARS) Colorimetric Assay Kit (Invitrogen, USA, Cat. No. E-BC-K298-M). Briefly, TBARS in serum reacted with thiobarbituric acid (TBA) under acidic and high-temperature conditions to form a pink adduct. The absorbance was measured at 535 nm, and MDA concentration was calculated from a standard curve, normalized to total protein content determined via the Bradford method (Bio-Rad, USA, Cat. No. 500–0210), and expressed as nmol/mg protein. The assay was performed according to the manufacturer’s instructions.

Serum was processed rapidly on ice, and mixed with pre-chilled 10% sulfosalicylic acid (SSA) at a ratio of 2:1. After incubation on ice for 10 min, acid-induced protein precipitation was completed [17]. No exogenous antioxidants were added; however, whole-process low-temperature handling combined with SSA acidification has been widely validated as a reliable approach to stabilize GSH in biological samples. GSH concentration was measured using the Reduced GSH Colorimetric Assay Kit (Elabscience Biotechnology Co., Ltd., China, Cat. No. E-BC-K030-M) based on the DTNB method at 412 nm, normalized by serum total protein content determined via the Bradford method (Bio-Rad, USA, Cat. No. 500–0210), and expressed as nmol/mg protein.

### Pulmonary function testing

Pulmonary function was assessed using a spirometer (Jaeger, Germany). Forced expiratory volume in one second (FEV_1_) and forced vital capacity (FVC) were measured. The percentage of predicted FEV_1_ (FEV_1_% pred) and the FEV_1_/FVC were calculated.

### Anthropometric measurements

Body mass index (BMI) was calculated as weight in kilograms divided by the square of height in meters (kg/m^2^).

### Statistical analysis

A formal a priori power calculation was not performed for this exploratory, single-center study. The sample size was determined based on the availability of consecutive patients during the study period. Continuous variables were tested for normality using the Shapiro–Wilk test. For variables with a normal distribution in both groups, data are expressed as mean ± standard deviation (SD), and between-group comparisons were performed using the independent-samples t-test. For variables with non-normal distribution in at least one group, data are presented as median (interquartile range, IQR), and between-group comparisons were performed using the Mann–Whitney U test. Categorical variables were presented as numbers and percentages (n, %). The correlations between Vanin-1 and other continuous variables were evaluated using.

Spearman’s correlation analysis. ROC curve analysis was performed to assess the diagnostic accuracy of Vanin-1. The optimal cutoff value was determined using the Youden index (J = sensitivity + specificity—1). The 95% confidence intervals for sensitivity and specificity at the optimal cutoff were calculated using the Wilson score interval method. All statistical analyses were performed using SPSS 31.0 (IBM Corp., Armonk, NY, USA). A two-sided *P*-value < 0.05 was considered statistically significant. The significance level was adjusted to α' = 0.05/11 ≈ 0.005, and only two-sided *P*-values < 0.005 were considered statistically significant after adjustment.

## Results

### Comparison of baseline characteristics

A total of 169 subjects were included in the final analysis, comprising 129 patients with asthma and 40 healthy controls. No significant differences were observed between the two groups regarding age, gender distribution, or BMI (Table 2).

**Table 2:** Baseline clinical and inflammatory characteristics of the study participants

	Asthmatic patients (*n* = 129)	Healthy controls (*n* = 40)	*P* value
Sex (M/F), n (%)	68 (52.7)/61 (47.3)	18 (45.0)/22 (55.0)	0.75
Age [median (IQR), years]	48.0 (34.0, 63.0)	50.0 (35.5, 65.0)	0.34
BMI (mean ± SD, kg/m^2^)	22.15 ± 3.08	22.45 ± 3.10	0.06
IL-4 [median (IQR), (pg/mL)]	247.21 (234.27, 272.06)	152.92 (124.08, 182.19)	< 0.001
IL-13 [median (IQR), (pg/mL)]	248.37 (230.20, 271.98)	136.45 (110.43, 163.64)	< 0.001
IL-17 [median (IQR), (pg/mL)]	753.00 (642.73, 829.46)	378.66(289.49, 464.06)	< 0.001
IFN-γ [median (IQR), (pg/mL)]	68.58 (58.17, 76.84)	148.67 (147.41, 150.67)	< 0.001
tIgE [median (IQR), (IU/mL)]]	151.00 (68.70, 320.73)	14.45 (12.93, 16.38)	< 0.001
Peripheral eosinophil count [median (IQR), 4 × 10^9^/L]	0.15 (0.08, 0.24)	0.12 (0.07, 0.60)	0.002
FEV_1_% pred [median (IQR)]	84.00 (76.35, 90.30)	87.65 (85.47, 90.60)	0.004
FEV_1_/FVC [median (IQR)]	80.30 (84.20, 89.70)	85.05 (84.45, 89.68)	0.003

Data are presented as n (%), mean ± SD, or median (IQR). Categorical variables were compared using the chi-square test. For continuous variables, the independent-samples t-test or the Mann–Whitney U test was applied based on data distribution. *P*-values are shown before multiple testing correction

### Differences in serum biomarkers

**Fig. 1:**
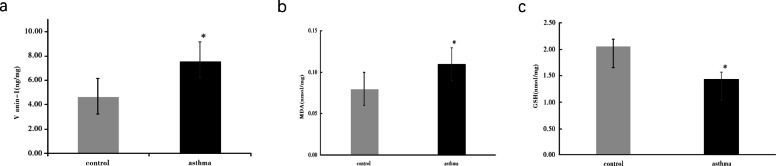
Comparison of serum Vanin-1, MDA, and GSH levels between healthy controls and asthmatic patients. **a** Vanin-1 levels are presented as mean ± standard deviation (SD) and were compared using the independent samples t-test; **b** MDA and **c** GSH levels are presented as median (interquartile range, IQR) and were compared using the Mann–Whitney U test. *P < 0.001 versus the control group

Vanin-1 levels were markedly higher in the asthma group (7.54 ± 1.62 ng/mL) than in the control group (4.59 ± 1.30 ng/mL, *P* < 0.001) (Fig. 1). MDA levels were significantly elevated in asthmatic patients [0.11 (0.09, 0.12) nmol/mg protein] compared to controls [0.08 (0.06, 0.10) nmol/mg protein, *P* < 0.001]. In contrast, GSH levels were substantially lower in the asthma group [1.44 (1.16, 1.57) nmol/mg protein] than in the control group [2.06 (1.65, 2.37) nmol/mg protein, *P* < 0.001]. Proinflammatory cytokine levels are summarized in Table 2. Serum levels of IL-13, IL-17, and IL-4 were significantly elevated in asthma patients compared to healthy controls (all *P* < 0.001) (Table 2). In contrast, IFN-γ levels were significantly lower in asthma patients compared with healthy subjects (*P* < 0.001) (Table 2). Additionally, the median serum tIgE level was significantly higher in the asthmatic group than in the control group (*P* < 0.001) (Table 2). Blood eosinophil counts were also significantly elevated in the asthmatic group compared to controls (*P* < 0.001) (Table 2).

### Differences in pulmonary function tests

Consistent with the asthma phenotype, patients exhibited significant impairment in pulmonary function. Both FEV_1_% and the FEV_1_/FVC were significantly lower in the asthmatic group than in the control group (both *P* < 0.001) (Table 2).

### Correlation analysis between serum Vanin-1 and other indicators

**Fig. 2:**
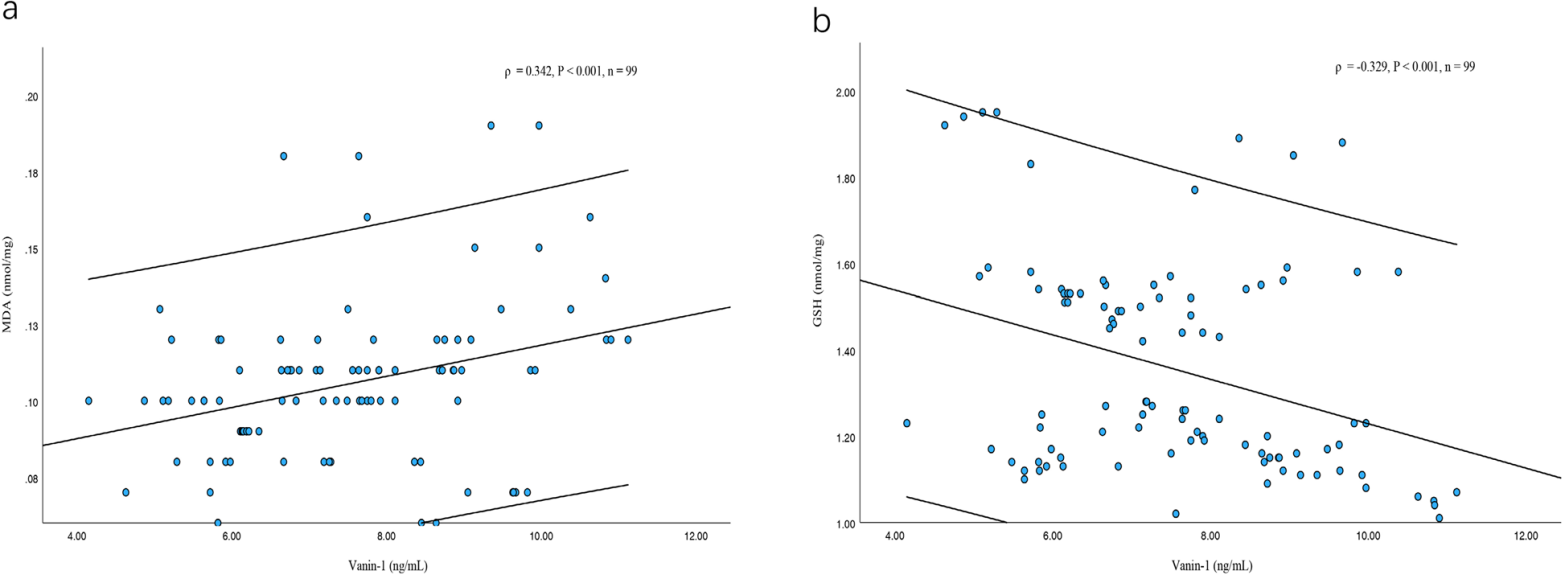
Correlation analysis between serum Vanin-1 levels and oxidative stress biomarkers. **a** Positive correlation between serum Vanin-1 and MDA levels (Spearman’s ρ = 0.342, *P* < 0.001). **b** Negative correlation between serum Vanin-1 and GSH levels (Spearman’s ρ = − 0.329, *P* < 0.001). Solid lines represent the regression lines; dashed lines represent the 95% confidence intervals

Spearman’s correlation analysis revealed that Vanin-1 levels were positively correlated with MDA concentrations (*ρ* = 0.342, 95% CI: 0.149–0.509, *P* < 0.001) and negatively correlated with GSH levels (*ρ* = − 0.329, 95% CI: − 0.499 to − 0.135, *P* < 0.001) (Fig. 2). However, no significant correlations were observed between Vanin-1 and age, sex, BMI, IL-4, IL-13, IL-17, IFN-γ, blood eosinophil count, tIgE, FEV_1_% predicted, or FEV_1_/FVC (Table 3).

**Table 3:** Correlations between serum Vanin-1 levels and other clinical indices (Spearman’s analysis)

Variable	Spearman correlation Coefficient (ρ)	*P* value
Sex (M/F), n (%)	0.082	0.421
Age [median (IQR), years]	0.095	0.367
BMI (mean ± SD, kg/m^2^)	0.124	0.228
IL-4 (pg/mL)	0.101	0.319
IL-13 (pg/mL)	0.005	0.962
IL-17 (pg/mL)	−0.151	0.136
IFN-γ (pg/mL)	0.110	0.279
sIgE (IU/mL)	0.028	0.780
Peripheral eosinophil count [median (IQR), 4 × 10^9^/L]	−0.132	0.194
FEV_1_% pred	0.053	0.601
FEV_1_/FVC	−0.118	0.244

### ROC curve analysis of serum Vanin-1 for asthma diagnosis

**Fig. 3:**
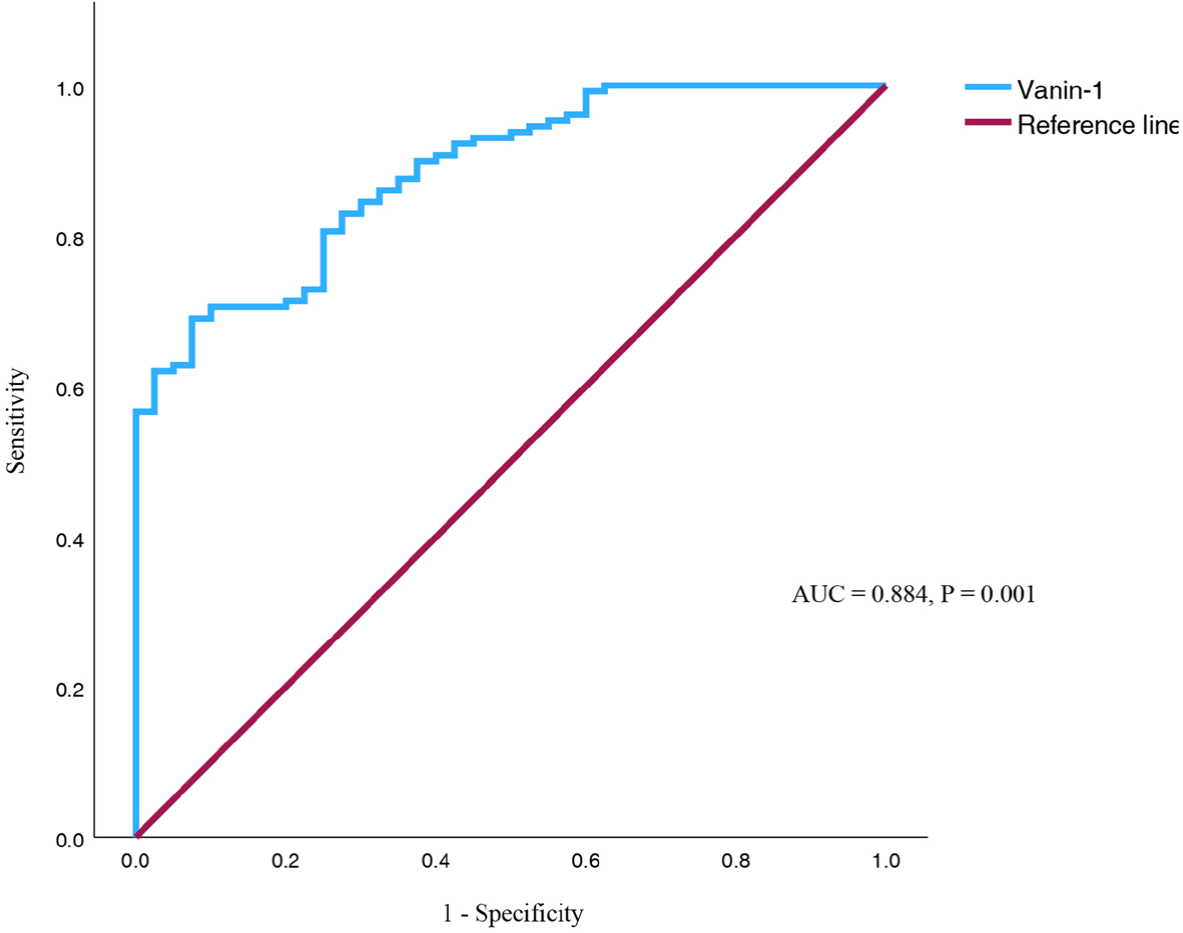
Receiver operating characteristic (ROC) curve analysis of serum Vanin-1 for diagnosing asthma. The area under the curve (AUC) was 0.884 (95% CI: 0.832–0.936, *P* < 0.001). The diagonal red line represents the line of no discrimination (AUC = 0.5)

ROC analysis was performed to assess the diagnostic performance of Vanin-1. Vanin-1 showed an excellent diagnostic performance for asthma, with an AUC of 0.884 (95% CI: 0.832–0.936, *P* < 0.001). The optimal cutoff value of Vanin-1 was 6.12 ng/mL, with a sensitivity of 69.0% (95% CI: 61.0%–77.0%), a specificity of 92.5% (95% CI: 84.3%–99.7%), and a maximum Youden’s index of 0.615 (Fig. 3).

## Discussion

Our study demonstrates that serum Vanin-1 levels are significantly elevated in patients with asthma compared with healthy controls, which is consistent with previous reports implicating Vanin-1 in asthma pathogenesis [8]. The significant correlations observed between serum Vanin-1 and established oxidative stress markers (MDA and GSH) provide clinical support for its biological role as a pantetheinase involved in oxidative stress regulation [18], suggesting that Vanin-1 may act as an oxidative stress sensor in asthma pathophysiology.

Free radicals, particularly reactive oxygen species (ROS), are pivotal drivers of oxidative stress and contribute to endothelial dysfunction in asthma. In asthmatic airways, chronic inflammation triggers excessive ROS production from activated inflammatory cells (e.g., neutrophils), dysregulated NADPH oxidase (Nox) activity, mitochondrial dysfunction, and environmental exposures such as air pollution [19]. This overwhelms endogenous antioxidant defenses (e.g., glutathione), creating an imbalance between ROS generation and scavenging capacity [20]. Consequently, ROS induce oxidative damage to lipids, proteins, and DNA, amplifying airway inflammation, mucus hypersecretion, bronchial hyperresponsiveness, and structural remodeling [19]. Critically, oxidative stress extends beyond the airways to impair vascular endothelial function. ROS directly reduce nitric oxide (NO) bioavailability—via eNOS uncoupling or reaction with superoxide—and promote endothelial injury, initiating a vicious cycle in which endothelial dysfunction further exacerbates vascular oxidative stress and inflammation [21]. Although asthma is primarily an airway disease, emerging evidence links its chronic inflammatory and oxidative milieu to systemic vascular consequences. For instance, asthma-associated oxidative stress may contribute to endothelial dysfunction underlying comorbidities such as accelerated atherosclerosis, highlighting shared pathophysiological pathways between the airway and vascular compartments [22]. This interplay underscores oxidative stress as both a local driver of airway pathology and a potential systemic mediator of vascular complications in asthma.

Within the context of this intertwined oxidative stress and immune dysregulation in asthma, we also evaluated serum levels of key immune effector cytokines. Patients with asthma exhibited marked alterations in Th2-associated cytokines (IL-4, IL-13), the Th17 cytokine (IL-17), and the Th1 cytokine IFN-γ. Specifically, IL-4, IL-13, and IL-17 levels were significantly elevated, whereas IFN-γ was reduced-consistent with the established immune dysregulation paradigm in asthma, characterized by Th2-polarized and Th17-mediated inflammation. However, no significant correlations were detected between Vanin-1 and any of these cytokines [23–25].

Although the absence of correlation may reflect the heterogeneity of asthma, where different endotypes exhibit distinct immune and oxidative stress profiles, and Vanin-1 may be more closely linked to oxidative stress in certain patient subsets rather than broadly associated with cytokine dysregulation, we acknowledge that several potential confounding variables known to influence oxidative stress, immune responses, and overall asthma phenotype were not systematically measured or adjusted for in our analysis. These include detailed asthma endotype stratification (e.g., T2-high vs. T2-low based on biomarker thresholds), use of biologic therapies, smoking or vaping status, occupational exposures, body mass index (given that obesity modifies asthma and oxidative stress), recent respiratory infections, and the presence of common comorbidities such as allergic rhinitis or gastroesophageal reflux disease (GERD). The absence of these data limits our ability to determine whether elevated Vanin-1 is specifically driven by asthma pathophysiology or is also influenced by these external or comorbid factors.

Serum Vanin-1 demonstrates strong diagnostic performance (AUC 0.884), with high specificity (92.5%) and acceptable sensitivity (69.0%), suggesting its potential as a complementary biomarker. Unlike classical markers such as blood eosinophils, which reflect type 2 inflammation, Vanin-1 appears to capture a distinct dimension of systemic oxidative stress. The lack of correlation observed between Vanin-1 and eosinophil counts in our study further supports its potential complementary value. Therefore, incorporating Vanin-1 into a multi-parameter panel that includes inflammatory indicators could enable a more comprehensive pathological assessment of asthma, thereby aiding in refined endotype stratification and informing treatment strategies targeting oxidative stress pathways [26].

Several limitations of this study should be noted. First, we acknowledge a methodological limitation regarding sample size, statistical power and the fact that the biochemical analyses were not performed under blinded conditions. No formal a priori power calculation was performed for the primary endpoints during the study planning phase. The sample size was determined pragmatically by participant availability during the recruitment period. Although stringent inclusion and exclusion criteria were applied to enhance cohort homogeneity, the lack of a prospective power calculation increases the risk of type II error for negative results (e.g., the lack of correlation with cytokines) and underscores the need for validation in larger, prospectively designed populations. Second, the case–control design precludes causal inference. Future prospective or interventional studies are needed to determine whether Vanin-1 plays a direct role in asthma pathogenesis or serves as a secondary marker of oxidative stress. Third, the single-center recruitment and limited ethnic diversity may affect the generalizability of our findings. Validation in larger, multi-ethnic, multi-center cohorts is essential. Fourth, we measured only systemic Vanin-1; assessment of its expression in local airway samples (e.g., sputum, BALF, or biopsies) could provide further insight into its site-specific role. Moreover, in endothelial cells, arginase functions as an endogenous competitor of nitric oxide synthase (NOS) for L-arginine, reducing substrate availability for NOS and thereby decreasing nitric oxide (NO) bioavailability while increasing superoxide anion (O_2_^-^•) production. This further impairs NO-mediated vasodilation and promotes endothelial dysfunction [27]. Future research could further investigate whether Vanin-1 influences endothelial cell function by modulating free radical generation or antioxidant pathways. Fifth, mechanistic studies using animal models—as performed by Kavian et al. [8] and Ferreira et al. [28] in other diseases-are warranted to elucidate how Vanin-1 modulates oxidative stress, inflammation, and airway remodeling in asthma. Such research could clarify its potential as a therapeutic target. Finally, we did not examine relationships between Vanin-1 and asthma severity, treatment response, or exacerbation risk. Future studies should assess whether Vanin-1 levels vary with disease severity or predict treatment outcomes and exacerbations, which would enhance its utility as a clinical biomarker. Additionally, the TBARS assay used in this study has limitation. Although it provides a valuable measure of overall lipid peroxidation, it is not completely specific for MDA, as it reacts with various thiobarbituric acid-reactive substances. Therefore, our results should be interpreted as reflecting a general increase in lipid peroxidation rather than a precise quantification of MDA. Future studies using more specific techniques, such as HPLC-based MDA measurement, would help confirm these findings.

Notwithstanding these limitations, our study identifies serum Vanin-1 as a novel biomarker in asthma that is closely linked to systemic oxidative stress. Its excellent diagnostic performance and distinct correlation profile suggest it may provide complementary information to existing inflammatory markers. Further mechanistic studies in model systems, alongside well-powered longitudinal clinical investigations, are warranted to validate its utility and elucidate its potential as a therapeutic target in asthma.

## Supplementary Information

Supplementary Material 1: Supplemental Table S1. Performance characteristics of the assay kits.

## Data Availability

The datasets generated and/or analyzed during the current study are not publicly available due to the protection of patient privacy and the restrictions of the local ethics committee, but are available from the corresponding author on reasonable request.
