# Supplementary material for: Serum Vanin-1: a potential diagnostic biomarker linked to oxidative stress imbalance in asthma
Source: BMC Pulm Med. 2026 May 4;26:285. doi: 10.1186/s12890-026-04319-7 (PMC13285491; doi:10.1186/s12890-026-04319-7)
Supplement: Supplementary file 1 — Supplementary Material 1: Supplemental Table S1. Performance characteristics of the assay kits. [file 12890_2026_4319_MOESM1_ESM.pptx]

## Slide 1
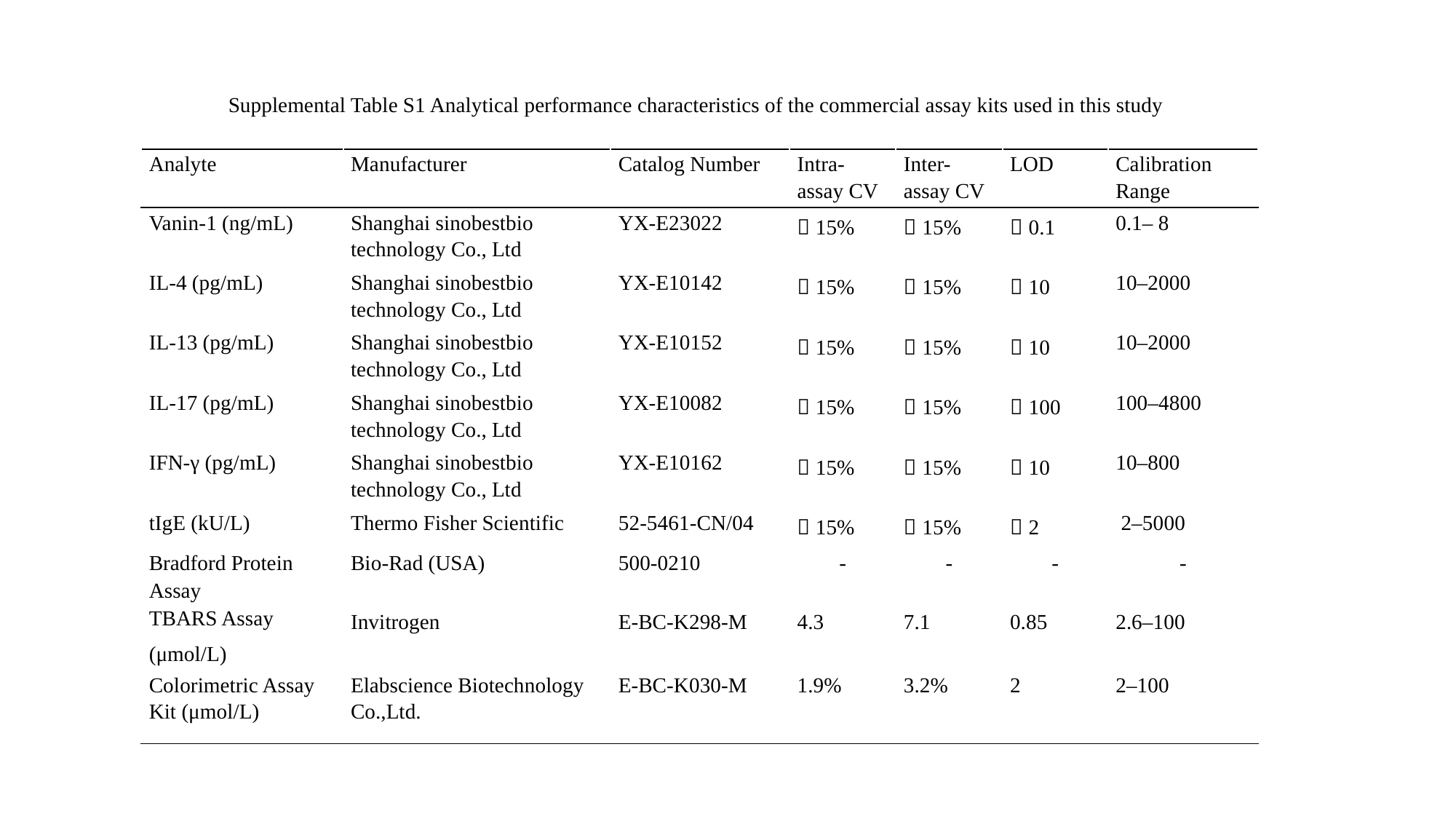

Supplemental Table S1 Analytical performance characteristics of the commercial assay kits used in this study
| Analyte | Manufacturer | Catalog Number | Intra-assay CV | Inter-assay CV | LOD | Calibration Range |
| --- | --- | --- | --- | --- | --- | --- |
| Vanin-1 (ng/mL) | Shanghai sinobestbio technology Co., Ltd | YX-E23022 | ＜15% | ＜15% | ＜0.1 | 0.1– 8 |
| IL-4 (pg/mL) | Shanghai sinobestbio technology Co., Ltd | YX-E10142 | ＜15% | ＜15% | ＜10 | 10–2000 |
| IL-13 (pg/mL) | Shanghai sinobestbio technology Co., Ltd | YX-E10152 | ＜15% | ＜15% | ＜10 | 10–2000 |
| IL-17 (pg/mL) | Shanghai sinobestbio technology Co., Ltd | YX-E10082 | ＜15% | ＜15% | ＜100 | 100–4800 |
| IFN-γ (pg/mL) | Shanghai sinobestbio technology Co., Ltd | YX-E10162 | ＜15% | ＜15% | ＜10 | 10–800 |
| tIgE (kU/L) | Thermo Fisher Scientific | 52-5461-CN/04 | ＜15% | ＜15% | ＜2 | 2–5000 |
| Bradford Protein Assay | Bio-Rad (USA) | 500-0210 | - | - | - | - |
| TBARS Assay (μmol/L) | Invitrogen | E-BC-K298-M | 4.3 | 7.1 | 0.85 | 2.6–100 |
| Colorimetric Assay Kit (μmol/L) | Elabscience Biotechnology Co.,Ltd. | E-BC-K030-M | 1.9% | 3.2% | 2 | 2–100 |
